# Mulberry branch fiber improved lipid metabolism and egg yolk fatty acid composition of laying hens via the enterohepatic axis

**DOI:** 10.1186/s40168-024-01788-y

**Published:** 2024-04-12

**Authors:** Hong Hu, Anjian Li, Changyou Shi, Liang Chen, Zelong Zhao, Xiaojian Yin, Qiang Zhang, Ying Huang, Hongbin Pan

**Affiliations:** 1 Yunnan Provincial Key Laboratory of Animal Nutrition and Feed Science, Faculty of Animal Science and Technology, Yunnan Agricultural University, Kunming, 650201 China; 2 University of Maryl and School of Medicine, Baltimore, MD 21228 USA; 3 State Key Laboratory of Animal Nutrition, Institute of Animal Sciences, Chinese Academy of Agriculture Sciences, Beijing, 100193 China; 4 Shanghai BIOZERON Biotechnology Co., Ltd, Shanghai, 201800 China; 5 WOD Poultry Research Institute, Beijing, 100193 China

**Keywords:** Multi-omics, Lipid metabolism, Egg quality, Laying hen, Short-chain fatty acids

## Abstract

**Background:**

The utilization of mulberry branch fiber (MF), the largest by-product of the sericulture industry, is an important issue. Supplementation with MF as a dietary fiber for poultry may serve as a useful application. However, little is known about the effects of MF on liver lipid metabolism and egg yolk fatty acid composition of laying hens and their underlying mechanisms. In this study, we performed a multi-omics investigation to explore the variations in liver lipid metabolism, egg yolk fatty acid composition, gut microbiota, and the associations among them induced by dietary MF in laying hens.

**Results:**

Dietary MF had no harmful effects on the laying performance or egg quality in laying hens. The enzyme activities associated with lipid metabolism in the liver were altered by the addition of 5% MF, resulting in reduced liver fat accumulation. Furthermore, dietary 5% MF induced the variation in the fatty acid profiles of egg yolk, and increased the polyunsaturated fatty acid (PUFA) content. We observed a significant reduction in the diversity of both gut bacteria and changes in their compositions after the addition of MF. Dietary MF significantly increased the abundance of genes involved in fatty acid biodegradation, and short-chain fatty acids biosynthesis in the gut microbiota of laying hens. The significant correlations were observed between the liver lipid metabolism enzyme activities of hepatic lipase, lipoprotein lipase, and total esterase with gut microbiota, including negative correlations with gut microbiota diversity, and multiple correlations with gut bacteria and viruses. Moreover, various correlations between the contents of PUFAs and monounsaturated fatty acids in egg yolk with the gut microbiota were obtained. Based on partial-least-squares path modeling integrated with the multi-omics datasets, we deduced the direct effects of liver enzyme activities and gut bacterial compositions on liver fat content and the roles of liver enzyme activities and gut bacterial diversity on egg yolk fatty acid composition.

**Conclusions:**

The results indicate that dietary MF is beneficial to laying hens as it reduces the liver fat and improves egg yolk fatty acid composition through the enterohepatic axis.

Video Abstract

**Graphical Abstract:**

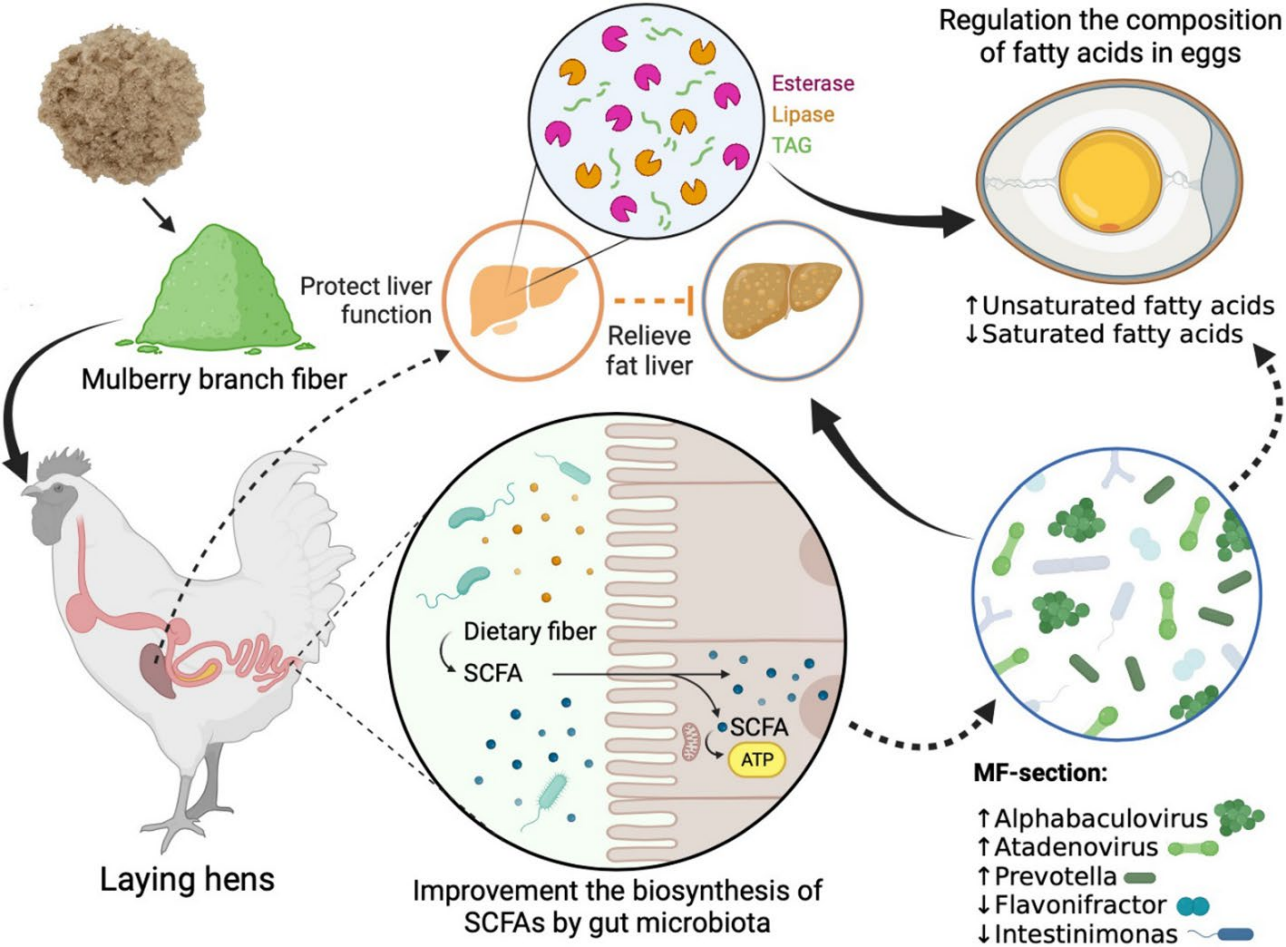

**Supplementary Information:**

The online version contains supplementary material available at 10.1186/s40168-024-01788-y.

## Background

The scale of breeding laying hens for food and economic purposes has increased continuously [1, 2]. Every laying hen has a peak egg production period, during which it undergoes high-intensity metabolism [3]. After this process, aged laying hens experience severe metabolic disorders, including accumulation of liver and abdominal fat [3], reduced antioxidant levels [4], and impaired reproductive system function [5]. These changes negatively affect the health of laying hens and impose restrictions on their production performance and egg quality [6]. To improve growth and promote the quality of cultured animals, many dietary fibers have been added to their diets [7]. Dietary fibers are complex polymers composed mainly of non-starch polysaccharides and lignin [8]. Adding an appropriate amount of dietary fiber to the diet can promote healthy development of the gastrointestinal tract [9], increase satiety [10], and be beneficial to the health and welfare of poultry [11]. It can also promote organ development, enhance digestive system function, and improve the production performance of poultry [12]. Several studies have reported the effects of dietary fiber on liver fat deposition and egg quality in poultry [13–15]; however, the underlying mechanisms remain unclear. In particular, there are few studies on the fatty acid composition of egg yolks from laying hens administered dietary fiber.

Mulberry is a small- to medium-sized tree belonging to the family Moraceae that produces sweet edible fruits [16]. In addition to being a food for humans, mulberry plants are an important food source for silkworms [17]. As the cradle of the sericulture industry, China possesses the most abundant mulberry germplasm resources and the largest mulberry planting area in the world [18]. Mulberry branches are one of the largest by-products of the sericulture industry, and their main component is mulberry branch fiber (MF) [19]. Natural cellulose is non-toxic, renewable, and degradable, and its utilization is an important way to utilize mulberry resources comprehensively [20]. The by-product of mulberry tree has been added to the diets of many domesticated animals, including dairy cows, sows, and lambs, to improve their growth and health status [21–23]. Moreover, the addition of fermented mulberry leaf powder to the diet of chickens positively affects their growth performance and meat quality [24]. Therefore, adding by-product of mulberry tree as a dietary fiber for poultry may serve as a useful application; however, its effects on laying hens need to be explored to expand its use.

As a high-nutrient food, eggs are one of the best sources of proteins and fatty acids for humans. With improvements in residents’ income and living standards, expectations and requirements for nutrition via egg products are increasing, and the demand for eggs has shifted from pursuing the satisfaction of quantity to improving quality [25]. The content of polyunsaturated fatty acids (PUFAs) in egg yolk is an important indicator to measure the nutritional value of eggs [26]. PUFAs have anti-atherosclerotic effects, relieve thrombosis, and reduce the risk of cardiovascular diseases [27]. Increased PUFA intake in the human body can alleviate lipid metabolism defects in patients with metabolic syndrome [28]. Thus, eggs with higher PUFAs in yolk have been considered “high quality” products and bring higher economic benefits to farmers.

The liver is the main site of fatty acid synthesis in chickens, unlike in mammals, where the adipose tissue plays a dominant role in lipogenesis [29]. During the egg-laying period, the estrogen level in hens increases significantly, prompting the liver to synthesize egg yolk precursors [30]. In addition, egg yolk fatty acids are synthesized by laying hens through their diet [31]. The intestine absorbs dietary lipids, which are then transported to the liver through the portal vein, and the liver subsequently synthesizes lipoproteins that transport lipids to the ovary, where they are incorporated into the yolk [32]. Therefore, the enterohepatic axis plays a crucial role in the synthesis of egg yolk fatty acids in laying hens; however, further research is required to understand the exact mechanisms involved.

Based on the above, we hypothesized that dietary fiber could improve the egg quality of laying hens by regulating yolk fatty acid composition via the enterohepatic axis. To explore this hypothesis, we designed a feeding experiment to determine whether dietary MF could enhance egg yolk fatty acid composition in laying hens. We evaluated the fatty acid profile of egg yolk, lipid metabolism in the liver of laying hens, and variations in the gut microbiota and microbial metabolites. The findings of this study provide a basis for the application of MF in the breeding of laying hens and expand our understanding of the mechanisms by which dietary fiber affects egg quality.

## Methods

### Mulberry branch fiber preparation and detection

Mulberry branches were selected, washed, sliced with a knife to a thickness of < 4 cm, and dried in an air blast oven at 55 °C for 24 h. The dried branches were milled into flour using a crusher and stored until use. The dietary fiber content (Total dietary fiber: TDF; Soluble dietary fiber: SDF; and Insoluble dietary fiber: IDF) of the prepared MF was analyzed by the ZKGX Research Institute of Chemical Technology (Beijing, China), according to the Chinese standard GB5009.88–2014. The protein and ash contents of the mulberry branches were detected using the GB/T 6432–2018 (Kjeldahl method) and GB/T 6438–20,007 (high temperature burning method) methods. The structure of the mulberry branch fibers was analyzed using scanning electron microscopy (Hitachi RegulusSU8100, Tokyo, Japan) at a voltage of 3 kV.

### Animals, experimental design, and sample collection

All procedures were approved by the Institutional Animal Care and Use Committee of the Yunnan Agricultural University (approval number: 202207049). A total of 360 laying hens of the Peking Pink strain (YunLingGuangDaYukou Poultry Co., Ltd., Yunnan, China) at the age of 57 weeks with similar performance were randomly allocated to five groups (12 hens/replicate, 6 replicates/group) and fed a basic diet containing 0% (CK group), 2% (MF2 group), 3% (MF3 group), 4% (MF4 group), and 5% (MF5 group) mulberry branch powder. The TDF contents of MF in the MF2, MF3, MF4, and MF5 groups were 1.5%, 2.2%, 2.9%, and 3.6%, respectively. Each hen was provided with a 112 g diet per day and had free access to water based on the management procedure for breeding Peking Pink laying hens (Yukou Poultry Co., Ltd., Beijing, China). The basal corn-soybean meal diet (Table S1) was formulated to meet the requirements of Peking Pink laying hens (NYT33-2004). The hens were raised in an enclosed, ventilated conventional house with 16 h of light and 55% relative humidity on average. The experiment lasted 49 days, including a 7-day acclimation period and a 42-day experimental period. Two eggs from each replicate were collected at the end of the experiment to determine egg quality, and one egg from each replicate was collected to determine the egg yolk fatty acid composition. One laying hen from each replicate was sacrificed by exsanguination. The liver and cecum digesta were immediately transferred into liquid nitrogen and stored at − 80 °C for further analysis.

### Measurement of laying performance and egg quality

The body weight (BW) of the laying hens was determined weekly. Egg number and weight were determined daily to calculate laying rate, egg weight, and egg mass. Feed efficiency was calculated in grams of feed consumed per day. Egg length, width, and eggshell thickness (air cell, equator, and sharp end) were determined using vernier calipers. Eggshell strength was determined using an egg force analyzer (Robotmation Model-III; Tokyo, Japan). The haugh unit, albumen height, yolk color, and yolk ratio were determined using an egg quality analyzer (Robotmation EMT-7300; Tokyo, Japan). Total lipids in the egg yolks from the CK and MF5 groups were extracted using a combination of chloroform and methanol (2:1, v/v), and the fatty acid profile was obtained using gas chromatography according to the detailed procedure described by Ghasemi et al. (2022) [33]. Based on the gas chromatography peaks, the total saturated fatty acids (SFAs), monounsaturated fatty acids (MUFAs), and PUFAs in egg yolk were calculated.

### Measurement of lipid metabolism in the liver

Oil red staining was performed by Wuhan Servicebio Technology Co., Ltd. (China). The liver samples were fixed in 4% paraformaldehyde. The fixed liver samples were routinely processed, sectioned at 5 μm, and stained with oil red O staining. The stained sections were observed using an optical microscope, and photographs were taken. The ratio of positive area to tissue area was calculated and analyzed using Aipathwell software. Triglyceride (TAG), total cholesterol (TCH), and low-density lipoprotein cholesterol (LDL-C) levels were determined using commercial kits (Nanjing Jiancheng Bioengineering Institute, Nanjing, China) following the manufacturer’s protocols. Liver enzyme activities of fatty acid synthase (FAS), acetyl-CoA carboxylase (ACC), lipase (LIP), hepatic lipase (HTGL), lipoprotein lipase (LPL), and total esterase (TES) were assessed using commercial kits (Nanjing Jiancheng Bioengineering Institute, Nanjing, China) according to the manufacturer’s protocols.

### Metagenomic sequencing of gut microbiota

Total microbial DNA from the cecum digesta was extracted using a FastDNA Spin Kit for Feces (MP Biomedicals, CA, USA) according to the manufacturer’s instructions. The quality and concentration of the extracted DNA were evaluated by agarose gel electrophoresis (1.5%) and spectrophotometry (NanoDrop 1000, Thermo Fisher Scientific Inc., USA). All extracted DNA was stored at − 20 °C for further applications. A total amount of 1 μg DNA per sample was used as input material for the library preparation. Sequencing libraries were generated using NEBNext® Ultra™ DNA Library Prep Kit for Illumina (NEB, USA) following the manufacturer’s recommendations, and index codes were added to help identify each sample. Briefly, the DNA sample was fragmented by sonication to a size of 350 bp, and the DNA fragments were then end-polished, A-tailed, and ligated with a full-length adaptor for Illumina sequencing with further PCR amplification. The PCR products were purified (AMPure XP system) and libraries were analyzed for size distribution using an Agilent 2100 Bioanalyzer. Metagenomic sequencing was performed using an Illumina NovaSeq 6000 platform. One sample with sterile water was used as the control for metagenomic library preparation and sequencing. After sequencing, raw data of low quality (phred score lower than 30, containing ambiguous bases and sequence length shorter than 150 bp) were removed using the NGS QC Toolkit to generate clean data before performing bioinformatics analysis [34].

Microbial community composition was determined using Kraken2, which maps metagenomic reads against a catalog of genomes currently spanning bacterial and viral phylogenies [35]. All the parameters of Kraken2 utilized the default settings. Taxonomic classifications at the phylum to species levels, as well as their corresponding read numbers, were obtained. The results were classified into bacteria and viruses according to their taxonomy, and the relative abundance of each taxon group was calculated. Two abundance tables for bacteria and viruses were obtained for further statistical analysis. Before functional annotation, metagenomic reads were assembled into contigs using MEGAHIT with default parameters [36]. After assembly, only the contigs longer than 500 bp were used for further analysis. Potential functional genes were predicted from contigs using MetaGeneMark with default parameters [37]. CD-hit was used to remove redundant sequences from the samples [38]. In each sample, clean reads were mapped back to the predicted genes using BBMap to obtain an accurate abundance of each gene [39]. For annotation of functional genes, the predicted genes were mapped to the KEGG database using BLASTP with an *e* value ≤ 1 × 10^−5^.

### Targeted metabolome of gut short-chain fatty acids and amino acids

All standards for short-chain fatty acids (SCFAs) and amino acids (AAs) were obtained from Sigma-Aldrich (St. Louis, MO, USA). To perform the targeted metabolome analysis, the caecum digesta was resuspended in liquid nitrogen and then added to water by thorough vortexing as the diluted sample. Then, 50 μL of the suspension was taken and homogenized by vortexing thoroughly with 200 μL of acetonitrile/methanol (1:1), which contained mixed internal standards. Next, the homogenate was put on ice for 30 min. After that, the sample was centrifuged at 12,000 rpm for 10 min. An ultrahigh performance liquid chromatograph coupled to tandem mass spectrometry (UHPLC–MS/MS) system (ExionLC™ AD UHPLC-QTRAP 6500 + , AB SCIEX Corp., Boston, MA, USA) was used to quantitate SCFAs and AAs. Separation was performed on an ACQUITY UPLC BEH Amide column (2.1 mm × 100 mm, 1.7 μm) which was maintained at 50 °C. The mobile phase, consisting of 0.1% formic acid in 5 mM ammonium acetate (solvent A) and 0.1% formic acid in acetonitrile (solvent B), was delivered at a flow rate of 0.30 mL/min. The solvent gradient was set as follows: initial 80% B, 0.5 min; 80–70% B, 2 min; 70–45% B, 4 min; 45–80% B, 6.01 min; 80% B, 9 min. The mass spectrometer was operated in positive multiple reaction mode. Parameters were as follows: IonSpray voltage (5500 V), Curtain gas (35 psi), Ion source temp (550 °C), Ion source gas of 1 and 2 (50 and 60 psi).

### Statistical analyses

All statistical analyses were performed using the R v4.2.2 platform. Differences in the contents of fatty acids in egg yolk and microbial metabolites in caecum digesta between the egg yolk of CK and MF5 groups were compared using Student’s *t* test. Meanwhile, principal coordinates analysis (PCoA) and the adonis test were performed (“vegan” package) to evaluate the differences in the fatty acid profile of egg yolk and microbial metabolites in caecum digesta of CK and MF5 individuals. Variations in the indices of liver lipid metabolism (biogeochemical indices and enzyme activities) were explored using Tukey’s HSD test (“multcomp” package). The alpha diversities (Chao1 and Shannon) of gut bacterial and viral communities were calculated, and differences between different groups were compared using Tukey’s HSD test. The Bray–Curtis distances of gut bacteria, viruses, and function structures among different samples were calculated, and then, principal coordinate analysis (PCoA) and adonis tests were performed. Tukey’s HSD test was also performed to compare the differences in the Bray–Curtis distance and mainly bacteria, viruses, and functional terms. To visualize the co-occurrence patterns (“ggraph” package), Pearson correlations were calculated based on the abundance of caecum bacteria, viruses, and functional terms. A correlation between two genes was considered statistically robust if the correlation coefficient was > 0.8 and the Benjamini–Hochberg adjusted *p* value was < 0.05 (“LinkET” package). Correlations of gut microbiota, lipid metabolism indicators, and fatty acid composition of egg yolk were investigated by Spearman correlation with the Benjamini–Hochberg adjusted *p* value (“psych” package). Finally, partial-least-squares path modeling (PLS-PM) was performed to quantify the underlying mechanism of dietary MF on liver metabolism and egg quality (“PLSPM” package).

## Results

### Characterization of MF

**Fig. 1:**
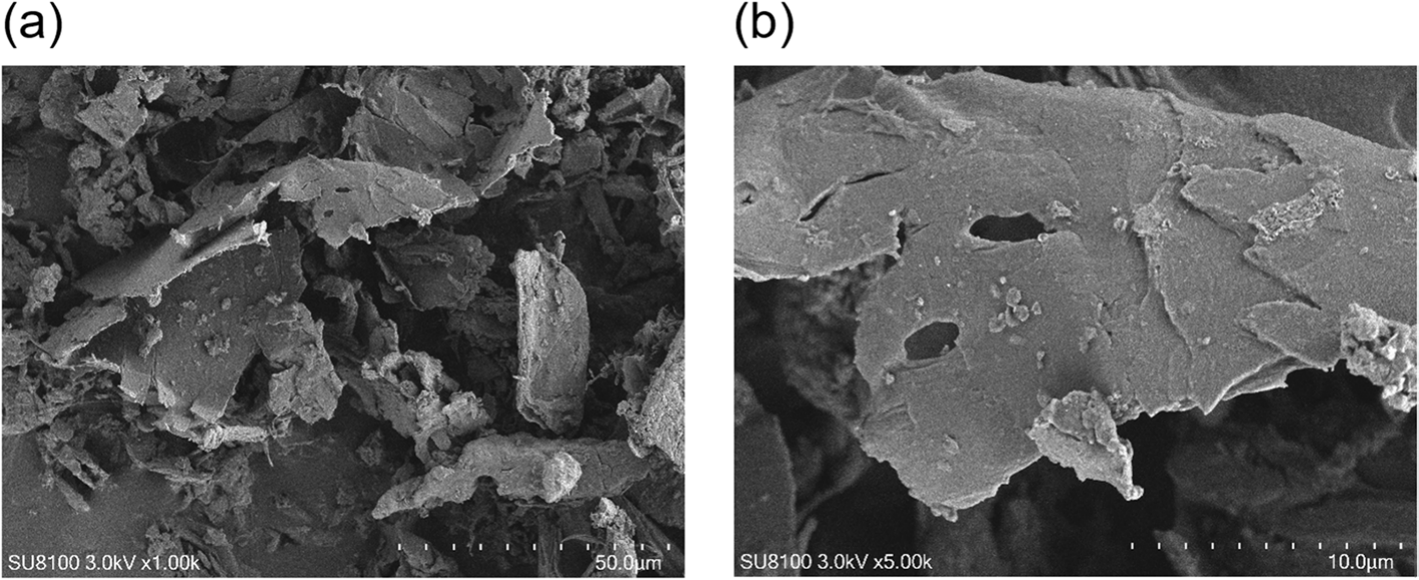
Scanning electron microscopy of MF. **a** 1000 × and **b** 5000 × magnifications

The TDF content of MF was 72.86%, with 72.84% IDF and 0.02% SDF (Table S2). The protein and ash levels were 4.43% and 11.18%, respectively (Table S2). As shown in Fig. 1, the fibers showed an amorphous structure. The MF had flaky layered structure structures with rough surface in scanning electron microscopy analyses.

### Laying performance

Dietary MF had no detectable influence on the final BW, laying rate, or feed efficiency (*p* > 0.05, Table 1). However, dietary MF had significant quadratic effects on the laying rate (*p* < 0.05, Table 1).

**Table 1:** Effect of the dietary MF on the laying performance

Indexes	CK	MF2	MF3	MF4	MF5	SEM	*P* value	Linear *P* value	Quadratic *P* value
Final BW, kg	2.00	1.96	1.92	1.94	1.94	0.10	0.144	0.061	0.087
Laying rate, %	89.62	84.85	87.10	86.31	88.59	0.65	0.142	0.891	0.040
Egg mass, g/hen/day	56.13	53.36	55.37	54.53	56.47	0.44	0.153	0.532	0.071
Feed efficiency, g/g	2.03	2.14	2.05	2.09	2.01	0.018	0.130	0.509	0.079

### Egg quality

Dietary MF did not significantly affect the average egg weight, egg shape index, eggshell thickness, eggshell strength, albumen height, Haugh units, yolk weight, yolk ratio, or yolk color score (*p* > 0.05, Table 2), but it showed significant linear effects on eggshell thickness (*p* < 0.05, Table 2).

**Table 2:** Effect of the dietary MF on the egg quality

Indexes	CK	MF2	MF3	MF4	MF5	SEM	*P* value	Linear *P* value	Quadratic *P* value
Average egg weight (g)	62.98	62.75	63.60	62.87	63.65	0.430	0.951	0.657	0.911
Egg shape index, %	1.27	1.30	1.31	1.29	1.29	0.006	0.338	0.343	0. 072
Eggshell thickness (mm)	0.36	0.35	0.35	0.33	0.34	0.004	0.106	0.024	0.264
Eggshell strength (N)	3.84	3.70	3.86	3.65	3.68	0.092	0.935	0.595	0.969
Albumen height (mm)	5.78	5.81	6.08	6.33	6.18	0.155	0.155	0.256	0.783
Yolk color score	7.57	7.57	7.57	7.57	7.57	0.090	0.370	0.532	0.537
Haugh units	70.72	74.21	75.68	77.45	75.53	1.293	0.581	0.180	0.348
Yolk weight (g)	17.49	17.51	17.15	17.17	17.33	0.123	0.843	0.472	0.545
Yolk ratio	0.28	0.26	0.27	0.27	0.27	0.005	0.820	0.954	0.524

### Lipid metabolism in the liver

**Fig. 2:**
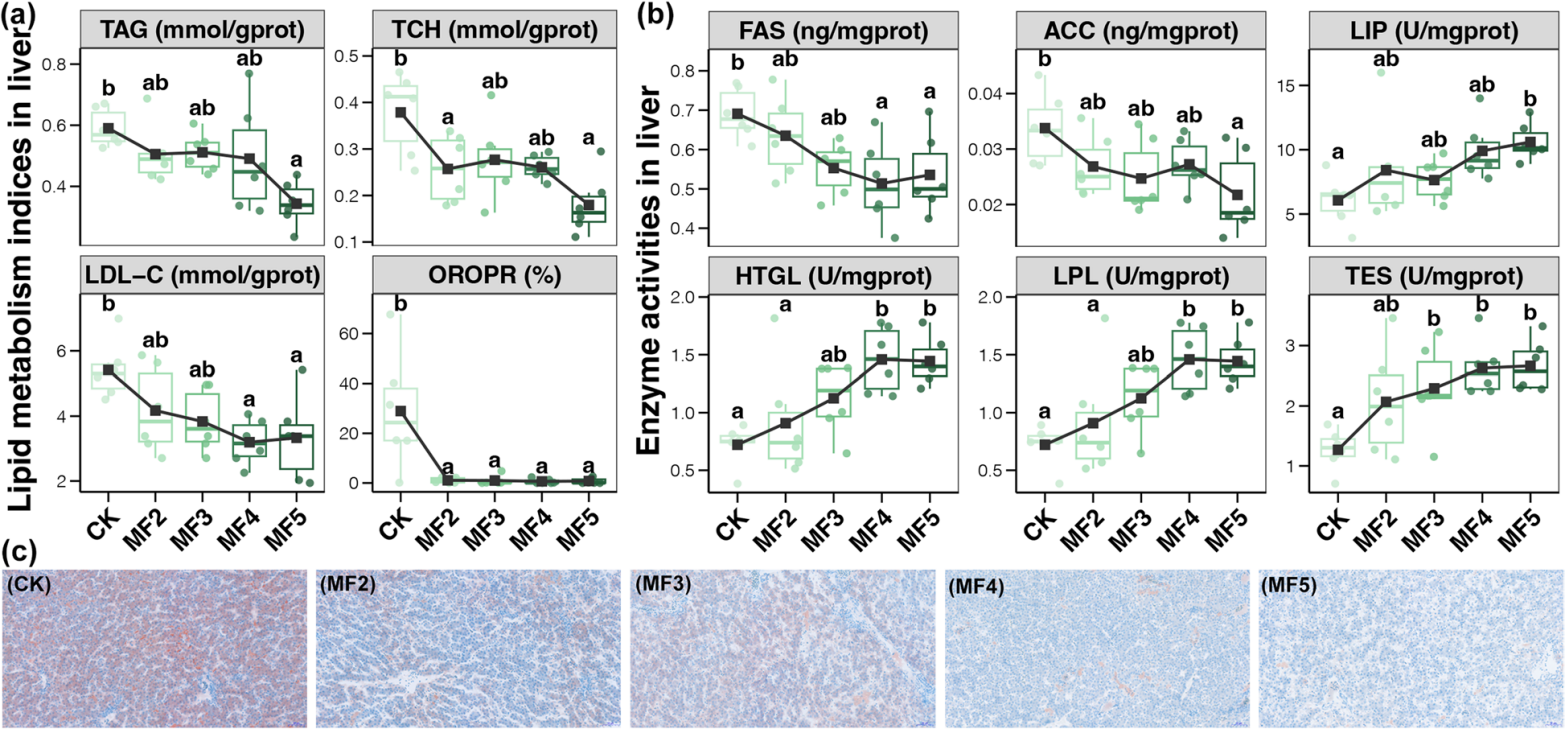
Differences in the liver fat indices (**a**) and enzyme activities related to lipid metabolism (**b**) among laying hens with and without dietary MF. Different lowercase letters in each box of the same subfigure represent significant differences among laying hens in different groups (Tukey’s HSD test, *p* < 0.05). **c** Light micrographs of Oil red stain from liver of laying hens with MF addition compared to the CK samples

Dietary 5% MF significantly reduced the liver fat content, including TAG, TCH, and LDL-C, of laying hens, and this reduction increased with an increase in the amount of MF added (Tukey’s HSD test, *p* < 0.05, Fig. 2a). Meanwhile, we also observed a remarkable decrease in the positive ratio of oil red staining in the liver of laying hens with MF addition compared with the CK samples (Tukey’s HSD test, *p* < 0.05, Fig. 2 a and c). Moreover, dietary 5% MF also reduced the activities of enzymes related to fatty acid synthesis (FAS and ACC), but increased the activities of enzymes related to lipid decomposition (including LIP, HTGL, LPL, and TES) in the liver of laying hens (Tukey’s HSD test, *p* < 0.05, Fig. 2b). The changes in enzyme activities of the liver also exhibited a significant dose-dependent relationship (Fig. 2b). These results suggested dietary MF could reduce liver fat accumulation to effectively control the risk of fatty liver in laying hens.

### Egg yolk fatty acid composition

**Fig. 3:**
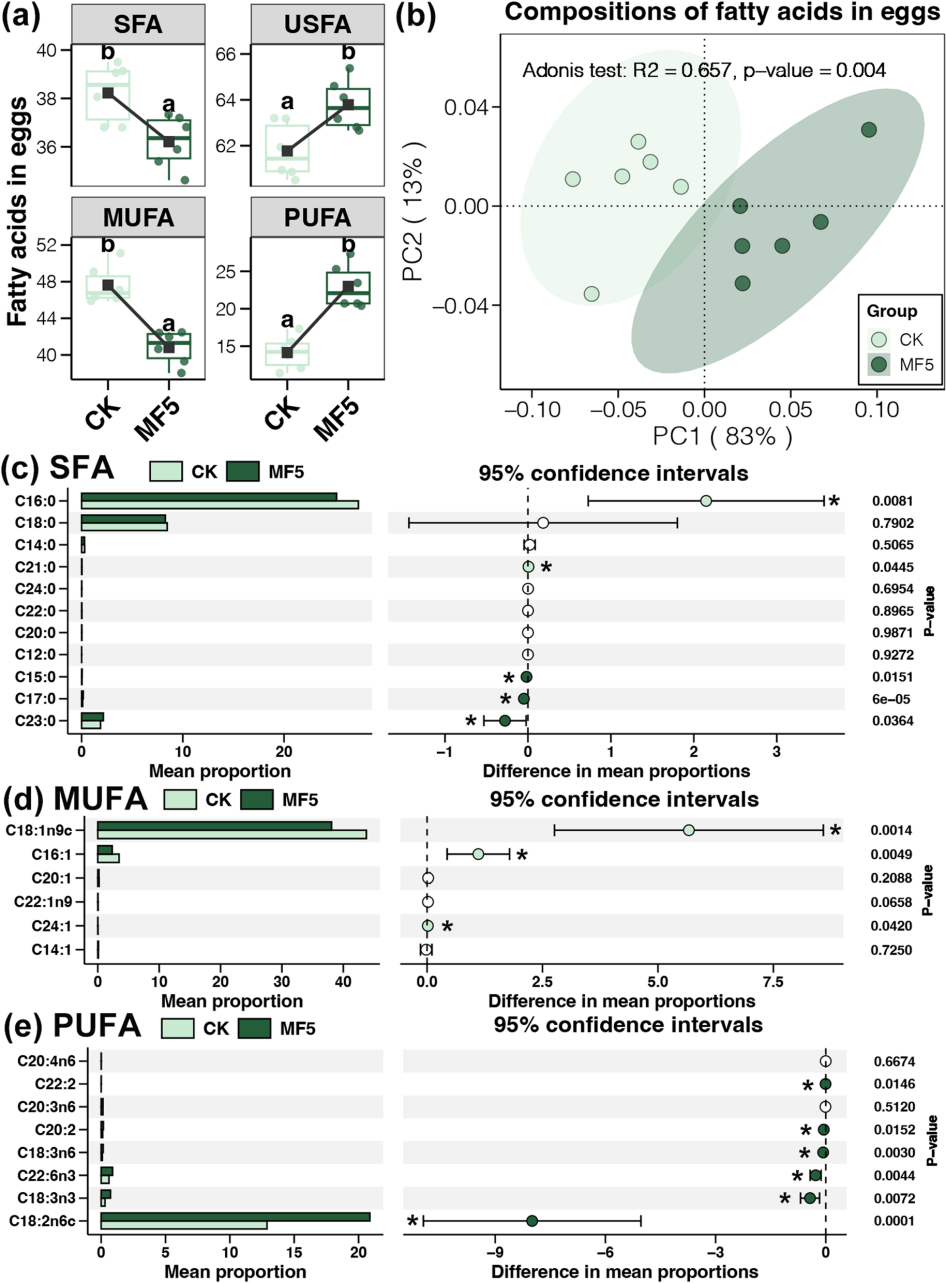
**a** Differences in the fatty acid contents between the egg yolk of the CK and MF5 groups. **b** Principal coordinate analysis (PCoA) of the fatty acid profiles in egg yolk of the CK and MF5 groups based on the Bray–Curtis distance. Differences in the contents of SFAs (**c**), MUFAs (**d**), and PUFAs (**e**) between the egg yolk of the CK and MF5 groups. * represents significant differences between laying hens of the CK and MF5 groups (Student’s *t* test, *p* < 0.05)

For egg yolk fatty acid composition, 5% dietary MF (MF5 group) significantly decreased and increased the contents of total SFA and total unsaturated fatty acids (USFA), respectively, in egg yolks compared with the CK group (Student’s *t* test, *p* < 0.05, Fig. 3a). Moreover, the increase in total USFA was mainly contributed by the enhanced PUFA in egg yolk (Student’s *t* test, *p* < 0.05, Fig. 3a). Furthermore, the compositions of fatty acids in egg yolk were found to be significantly varied between the MF5 and CK samples (adonis test, *p* < 0.05, Fig. 3b). In detail, 5% dietary MF significantly increased the concentrations of some SFAs (pentadecanoic acid, C15:0; heptadecanoic acid, C17:0; and tricosanoic acid, C23:0) in the egg yolk, whereas palmitic acid (C16:0) and heneicosanoic acid (C21:0) were significantly reduced (Student’s *t* test, *p* < 0.05, Fig. 3c). For the MUFAs, palmitoleic acid (C16:1), oleic acid (C18:1n9c), and nervonic acid (C24:1) levels were significantly lower in the MF5 samples than in their CK counterparts (Student’s *t* test, *p* < 0.05, Fig. 3d). In contrast, contents of multiple PUFAs (linoleic acid, C18:2n6c; α-linolenic acid, C18:3n3; γ-linolenic acid, C18:3n6; eicosadienoic acid, C20:2; docosadienoic acid, C22:2; and docosahexaenoic acid, C22:6n3) were significantly higher in the MF5 samples than those in the CK samples (Student’s *t* test, *p* < 0.05, Fig. 3e). These findings proved the effects of MF on egg quality involving the promotion of PUFA content in egg yolk.

### Variations in gut microbiota

**Fig. 4:**
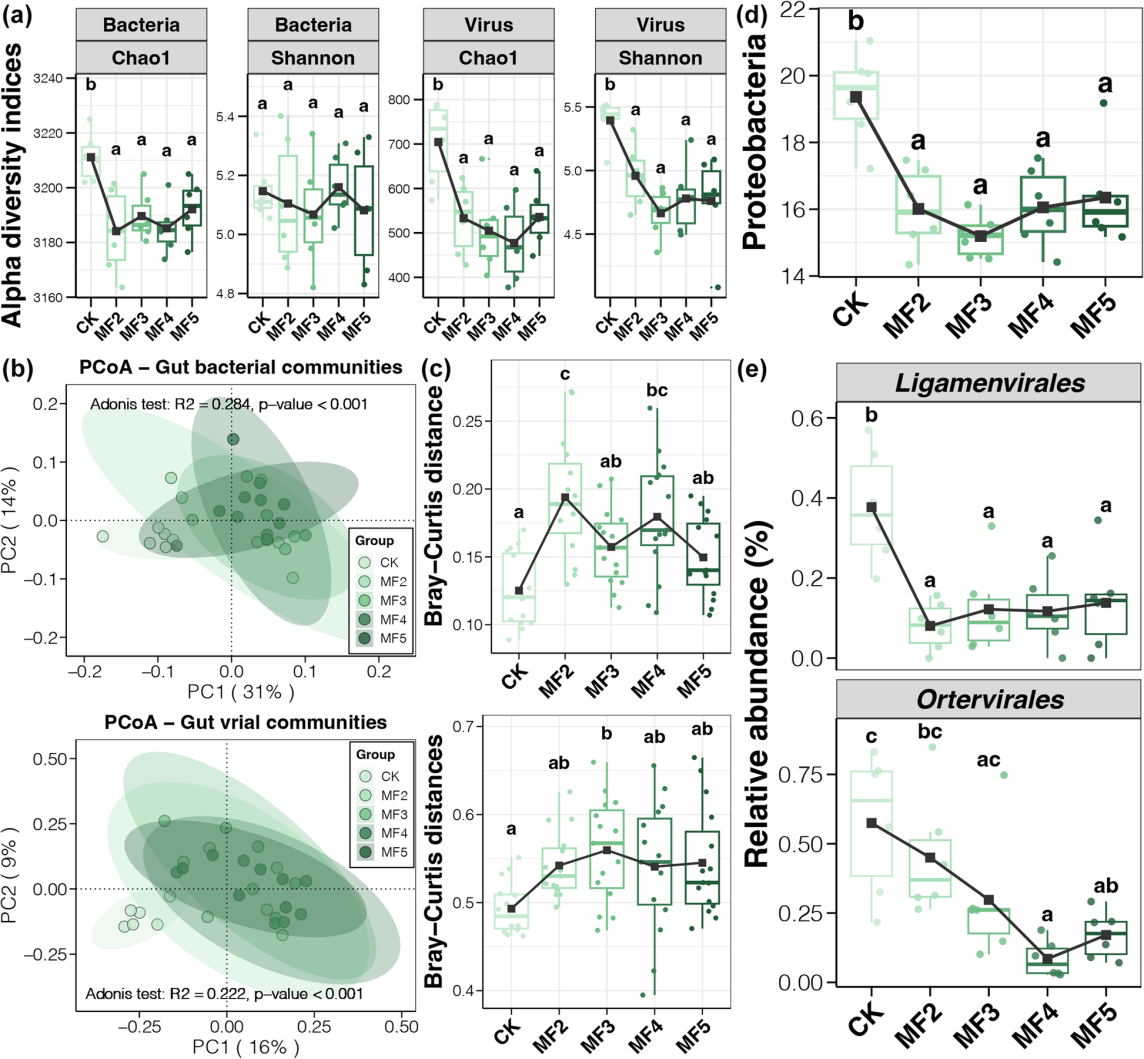
**a** Differences in the alpha diversity indices of gut bacterial and viral communities among laying hens with and without dietary MF. **b** PCoA and adonis test based on the Bray–Curtis distance of gut bacterial and viral communities among laying hens with and without dietary MF. **c** The difference in the Bray–Curtis distance of gut bacterial and viral communities among laying hens with and without dietary MF. **d** Variations in the relative abundance of Proteobacteria in the caecum digesta of laying hens with and without dietary MF. **e** Variations in the relative abundance of Ligamenvirales and Ortervirales in the caecum digesta of laying hens with and without dietary MF. Different lowercase letters in each box of the same subfigure represent significant differences among laying hens in different groups (Tukey’s HSD test, *p* < 0.05)

Based on the results obtained from the metagenomic sequencing, we observed a significant decrease in the Chao1 index of both gut bacterial and viral communities in laying hens with MF addition compared to those without MF addition (Tukey’s HSD test, *p* < 0.05, Fig. 4a). Meanwhile, a significantly lower Shannon index induced by dietary MF was also found for gut viral communities but not in bacterial communities (Tukey’s HSD test, *p* < 0.05, Fig. 4a). In addition, we also obtained significant variations in gut bacterial and viral communities in laying hens with and without dietary MF (adonis test, *p* < 0.05, Fig. 4b). Both gut bacterial and viral communities in samples of the CK group were separately clustered and disjointed from samples with dietary MF (MF2–MF5) (Fig. 4b). Moreover, we further observed increased intergroup variations in both gut bacterial and viral communities of laying hens with MF addition compared with the CK group (Tukey’s HSD test, *p* < 0.05, Fig. 4c). These findings indicated the apparent effects of dietary MF on the diversity and composition of gut microbiota of laying hens.

Bacteroidetes was the dominant bacterial phylum in the caecum digesta of laying hens, followed by Firmicutes, Proteobacteria, and Actinobacteria (Fig. S1). Among these main phyla of gut bacteria, only Proteobacteria was found to be significantly affected by the MF addition, showing a remarkable decrease in its relative abundance in caecum digesta of laying hens with dietary MF compared with the control (Tukey’s HSD test, *p* < 0.05, Fig. 4d). At the genus level, *Bacteroides* was the dominant bacterial genus, followed by *Barnesiella*, *Paenibacillus*, *Alistipes*, and *Faecalibacterium* (Fig. S2). Several bacterial genera were found to be significantly affected by dietary MF, expressed as reduced *Desulfovibrio*, *Flavonifractor*, *Intestinimonas*, and *Pseudomonas*, but enriched *Muribaculum* and *Prevotella* (Tukey’s HSD test, *p* < 0.05, Fig. S3). Among gut viruses, Caudovirales was the dominant order, followed by Herpesvirales (Fig. S4). Significant variations in the relative abundance of viruses were only found in two relatively rare orders, Ligamenvirales and Ortervirales, which were reduced after MF addition (Tukey’s HSD test, *p* < 0.05, Fig. 4e). Among viral genera, *Alphabaculovirus* was the most abundant, followed by *D3virus*, *Pandoravirus*, and *T4virus* (Fig. S5). Six viral genera were significantly affected by dietary MF, expressed by enriched *Alphabaculovirus*, *D3virus*, and *T4virus* but reduced *Cytomegalovirus*, *Mimivirus*, and *Varicellovirus* (Tukey’s HSD test, *p* < 0.05, Fig. S6).

### Variations in functions of gut microbiota

**Fig. 5:**
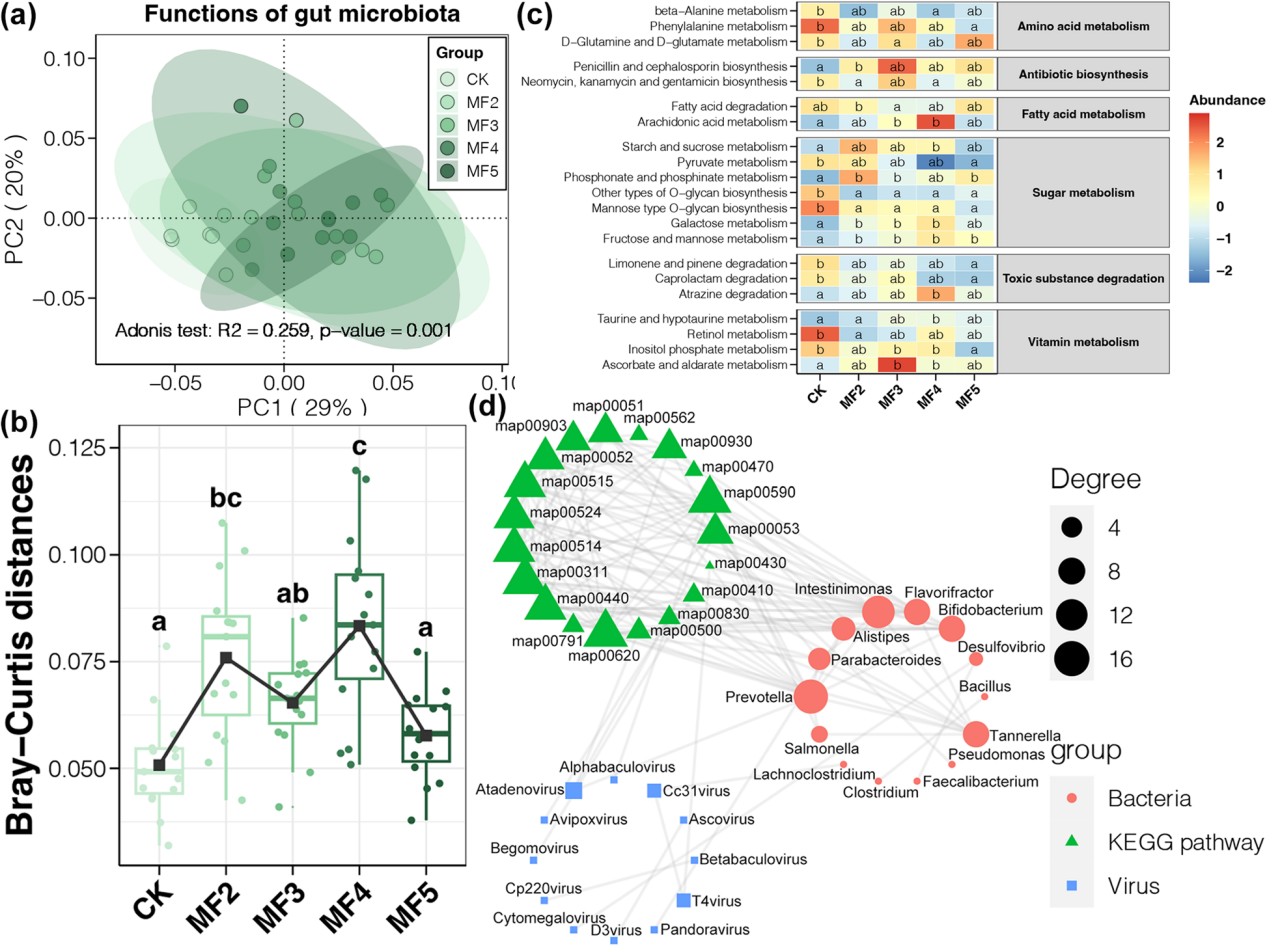
**a** PCoA and adonis test based on the Bray–Curtis distance of gut microbial functions among laying hens with and without dietary MF. **b** The difference in the Bray–Curtis distance of gut microbial functions among laying hens with and without dietary MF. **c** The heatmap shows gut microbial functional pathways with different abundances among laying hens with and without dietary MF. Different lowercase letters in each box of the same subfigure represent significant differences among laying hens in different groups (Tukey’s HSD test, *p* < 0.05). **d** The network shows correlations among functional pathways with bacterial and viral genera in the caecum digesta of laying hens with and without dietary MF

Similar to the composition of the gut microbiota, PCoA also showed separate clusters between laying hens with and without MF addition (adonis test, *p* < 0.05, Fig. 5a). Moreover, the intergroup variation in gut microbial functions was also increased by dietary MF (Tukey’s HSD test, *p* < 0.05, Fig. 5b). Based on the KEGG annotation, we observed that the abundance of several metabolism pathways in the caecum digesta of laying hens was significantly affected by dietary MF (Tukey’s HSD test, *p* < 0.05, Fig. 5c). Dietary MF reduced the ability of AA metabolism and O-glycan biosynthesis while improving the metabolism of main food (such as fatty acids and several saccharides). In addition, dietary MF strengthened the biosynthesis of some antibiotics (penicillin and cephalosporin), degradation of some toxic substances (atrazine), and metabolism of some vitamins (taurine and ascorbate), while restricting other pathways (antibiotics: neomycin, kanamycin, and gentamicin; toxic substances: limonene, pinene, and caprolactam; and vitamins: retinol and inositol phosphate). Moreover, we explored the potential relationships between the gut microbial compositions and these various functional pathways using the network analysis. Our results showed that the gut microbial functions were more closely connected to bacteria, with limited correlations to gut viruses and very few relationships involving bacteria, viruses, and functional pathways simultaneously (Fig. 5d). In particular, functional pathways that correlated to gut viruses were decreased in their abundance after MF addition, such as taurine and hypotaurine metabolism (map00430), beta-alanine metabolism (map00410), and retinol metabolism (map00830) (Fig. 5d).

### Metabolism of gut microbiota

**Fig. 6:**
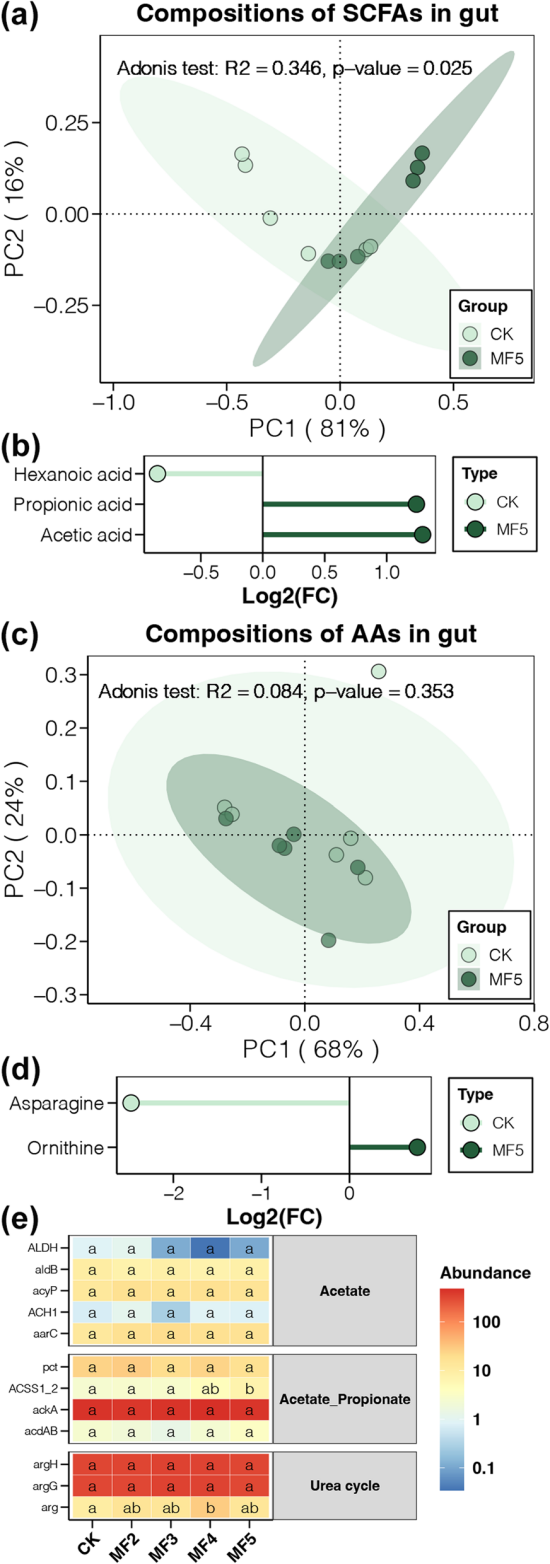
**a** PCoA and adonis test based on the Bray–Curtis distance of SCFA compositions in the caecum digesta between laying hens with and without dietary MF. **b** SCFAs with significantly different contents in the caecum digesta between laying hens with and without dietary MF. **c** PCoA and adonis test based on the Bray–Curtis distance of AA compositions in the caecum digesta between laying hens with and without dietary MF. **d** AAs with significantly different contents in the caecum digesta between laying hens with and without dietary MF. **e** The heatmap shows the abundance of genes related to the biosynthesis of SCFAs and the urea cycle in the caecum digesta of laying hens with and without dietary MF. Different lowercase letters in each box of the same subfigure represent significant differences between laying hens in different groups (Student’s *t* test, *p* < 0.05)

Based on the results of metagenomic sequencing, we measured the compositions of SCFAs and AAs in the caecum digesta of laying hens from the CK and MF5 groups using the targeted metabolome. Our results revealed significant variations in the compositions of SCFAs between the CK and MF groups, but not for AAs (Fig. 6 a and c). Furthermore, we detected significantly higher acetic acid and propionic acid in the caecum digesta of laying hens with MF addition than in CK individuals (Fig. 6b), indicating that dietary MF promoted the synthesis of SCFAs by gut microbiota. In contrast, the content of ornithine and asparagine significantly increased and decreased, respectively, in the caecum digesta of laying hens with MF addition compared with the CK group (Fig. 6d), suggesting the effect of dietary MF on the urea cycle. Subsequently, the genes responsible for the biosynthesis of acetic acid and propionic acid as well as key genes in the urea cycle from the metagenomic dataset were picked out. The results showed that the effects of dietary MF on the biosynthesis of SCFAs might mainly be associated with the more abundant *ACSS1_2* gene (Fig. 6e).

### Mechanisms of dietary MF to regulate laying hens

**Fig. 7:**
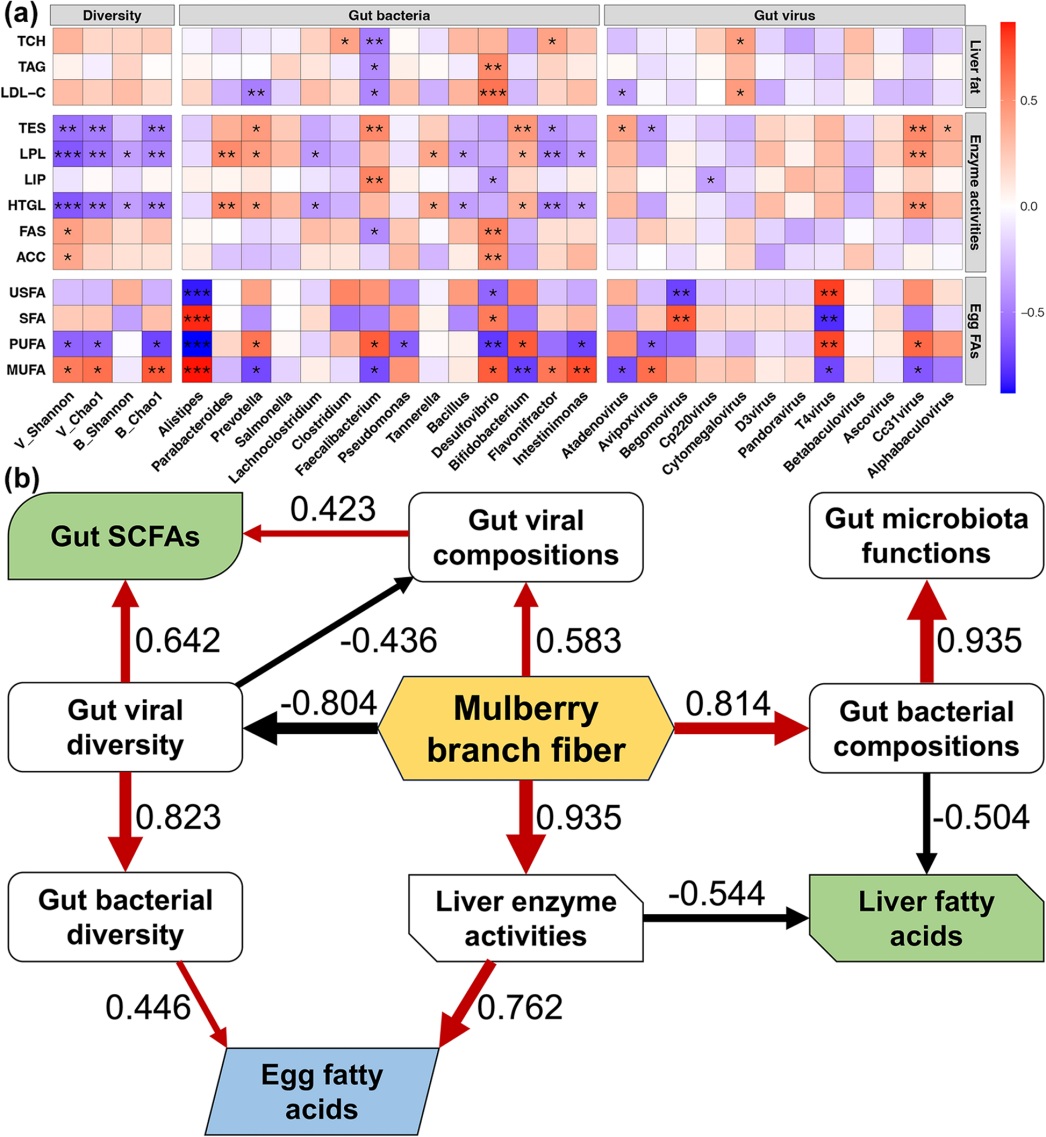
**a** Heatmap showing correlations of gut microbiota, lipid metabolism indicators and fatty acid composition of egg yolk. Color in each block represents the correlation coefficient. *, **, and *** represent the *p* value lower than 0.05, 0.01, and 0.001, respectively. **b** Partial least-squares path modeling showing the effects of dietary MF on the health state and egg quality of laying hens. Only paths with significant tests (*p* < 0.05) are shown in the plot. Positive and negative effects are shown by red and black lines, respectively. The line width was assigned according to the correlation coefficient

Correlations of gut microbiota, lipid metabolism indicators, and fatty acid composition of egg yolk were performed firstly (Fig. 7a). The correlations were found between the gut microbiota and liver fat contents, mainly involving the negative correlation with *Faecalibacterium* and positive correlation with *Desulfovibrio* and *Cytomegalovirus*. In addition, significant correlations were observed between the liver lipid metabolism related enzyme activities of TES, LPL, and HTGL with gut microbiota, including negative correlations with gut microbiota diversity, and multiple correlation with gut bacteria and virus. Moreover, various correlations between the contents of PUFA and MUFA in egg yolk with the gut microbiota were obtained. Furthermore, PLS-PM was performed to explore potential mechanisms of dietary MF to regulate the health state and egg quality of laying hens (Fig. 7b). Our results showed that dietary MF positively and negatively influenced the diversity and composition of gut viral communities in laying hens, respectively, which further regulated the biosynthesis of SCFAs. In addition, MF addition positively changed the gut bacterial composition and liver enzyme activities, which controlled the fatty acids in the liver of laying hens. Moreover, dietary MF improved the egg quality through the direct effects on liver enzyme activity and indirect effects on gut bacterial diversity.

## Discussion

Dietary fiber is a polysaccharide or lignin that cannot be digested and absorbed by the gastrointestinal tract or used to generate energy. Therefore, it was once considered an “nutrient-free substance” and has not received sufficient attention for a long time. With the in-depth development of nutrition and related sciences, dietary fiber which is recognized as the seventh category of nutrients by the nutritional community, has been found to play a significant physiological role for animals. The present study aimed to provide a basis for the application of MF in laying hens by investigating the effects of adding MF to the diet on the lipid metabolism, egg quality, and gut microbiota in the enterohepatic axis of laying hens. According to our results, dietary MF had no harmful effects on laying performance. Similarly, Walugembe et al. (2014), Wang et al. (2023), and Roberts et al. (2007) found that various dietary fibers (such as distillers dried grains with soluble, rice bran, wheat middlings, and soybean hulls) had no significant impact on layer hens [40–42]. The present results demonstrated that there were no detrimental effects of adding MF to the diet for layer hens.

Fatty liver is a common metabolic disorder in laying hens that can lead to sudden death and decreased egg production [43, 44]. During the lipid metabolism disorders progression, the excess circulating free fatty acids from adipose tissue can be stored in the liver, resulting in an imbalanced lipid homeostasis [45]. Nutritional supplements have been recommended to reduce the incidence of lipid metabolism disorders in laying hens [46]. An increase in TAG content in the liver is one of the most apparent features of fatty liver disease [47]. In this study, we observed a significant decrease in TAG content in the liver of laying hens with dietary MF, indicating the amelioration of liver lipid metabolism disorders by MF addition. In addition, we observed significantly decrees in the activities of ACC and FAS in liver of laying hens fed the MF diet. ACC is a biotin-containing enzyme that catalyzes the carboxylation of acetyl-CoA to malonyl-CoA, which is the rate-limiting step in fatty acid synthesis [48]. FAS is a multifunctional protein that catalyzes the synthesis of palmitate from acetyl-CoA and malonyl-CoA into long-chain saturated fatty acids in the presence of NADPH [49]. In contrast, the activities of several lipases, the rate-limiting step for the removal of TAGs from the circulation [50], increased in liver of laying hens fed by MF. Our results indicated that the addition of MF regulated the liver fatty acid metabolism of laying hens, limiting the synthesis of fatty acids in the liver and promoting their decomposition and utilization.

Except for reducing the risk of liver lipid metabolism disorders, dietary MF also showed effects on fatty acid composition of egg yolk mediated by the variations in liver lipid metabolism in laying hens. With the advance in research, the multiple nutritional strategies (including energy-to-egg ratio, different sources of fat, minerals, etc.) have been developed to improve egg quality [51]. The PUFA content in egg yolk has been linked to several health benefits for humans that consume eggs, including a decreased risk of heart disease, inhibition of cancer growth, and improved lipid health indices [52–54]. Egg yolks from hens fed with different diets have different fatty acid compositions. Eggs laid by hens fed diets containing fish oil have higher levels of docosahexaenoic acid and eicosapentaenoic acid than those fed with soybean oil [55]. The total n–3 fatty acids, docosahexaenoic acid, and docosahexaenoic acid:arachidonic acid ratios are higher in eggs from hens fed with n–3 PUFA-rich diets [56]. However, research regarding the influence of MF on egg yolk fatty acid profiles in laying hens remains scarce. The results of our study suggested increased PUFA content in the egg yolk of laying hens with dietary MF. Yolk fats are synthesized in the hen’s liver and deposited in the yolk through serum via triacylglycerol-rich very low-density lipoprotein [57]. This information was consistent with our results on the direct effects of liver enzyme activity on the fatty acid compositions of egg yolk in laying hens supplemented with MF.

Moreover, the addition of MF was found to alter the composition of gut bacteria in laying hens and enhanced their ability to decompose fatty acids in food. The intestinal microbiota of laying hens is involved in the modulation of nutrient utilization, the immune system, and the improvement of host health and production performance [58]. The gastrointestinal compartments of chickens are densely populated with complex microbial communities, including bacteria, fungi, archaea, protozoans, and viruses [59]. Compared with the large number of studies on intestinal bacteria, gut viral communities in poultry have been the subject of recent studies [60]. Gut viruses, mainly bacteriophages, can not only alter the composition of gut bacteria by infecting specific bacteria, but are also directly involved in the digestion and absorption of food via auxiliary metabolic genes carried by them [61, 62]. Dietary fiber cannot be digested or absorbed by the small intestine of poultry. However, it can be partially degraded by large intestine microorganisms, producing metabolic products such as SCFAs [63]. In this study, we investigated both gut bacterial and viral communities in laying hens at the same time using the metagenomic approach. SCFAs are important metabolites produced by the gut microbiota through the fermentation of nondigestible carbohydrates, including dietary fiber [64]. SCFAs have been implicated in glucose homeostasis, gut–brain communication, and gut epithelial and immune regulation which may reduce the risk of inflammatory diseases, type 2 diabetes, obesity, heart disease, and other conditions [65, 66]. In addition, SCFAs produced by the intestinal microbiota of poultry reduce the luminal pH and enhance calcium solubility, utilization, and deposition [67]. Multiple studies have demonstrated that the gut microbiota plays a significant role in modulating inflammatory and immune responses of their host, and that SCFAs are potential mediators of gut inflammation [68]. However, there is limited research on the relationship between gut viral communities and SCFA synthesis. In our study, we showed the potential relationship between gut viral communities and SCFA synthesis; however, further studies are needed to investigate this relationship and its implications for host health.

Finally, we explored the possible associations between variations in the gut microbiota and egg yolk fatty acid composition in laying hens. The gut microbiota of laying hens is a potential mediator for improving egg quality during the late phase of egg production [67]. Many studies suggested that providing an appropriate fiber source in the diets of nonruminants has gained considerable interest as a means to improve gastrointestinal tract (GIT) health, which is concomitant with a balanced microbiota [69, 70]. Similarly, our results indicated the connections between gut microbial structure and the physiological functions of hosts, and we observed the high egg quality of laying hens accompanied by reduced gut microbial richness. Furthermore, the diversity of gut bacteria directly affected the egg quality, which was regulated by the diversity of gut viruses altered by MF supplementation in the present study. The gut virome is composed of eukaryotic viruses and bacteriophages, which can replicate in gut bacteria. Previous studies have reported that viruses in the gut can shape the gut microbiome and that diet may influence the presence of viruses in the gut [71, 72]. However, the interaction between the gut virome and microbiome is a subject of ongoing research. It is important to note that although viruses can have detrimental effects on animal health, some viral interactions with the gut microbiota can be beneficial. Therefore, further research is required to explore the mechanisms underlying the relationship between gut microbial diversity and host physiological functions.

## Conclusions

Here, we showed the beneficial effects of dietary MF on liver lipid metabolism and egg quality in laying hens and their underlying mechanisms using a multi-omics approach. Dietary MF showed no obvious influence on the laying performance of tested hens with the improvements of liver lipid metabolism and egg quality. First, MF addition significantly changed the gut microbiota composition of laying hens, with reduced diversity of both gut bacteria and viruses and selective enrichment or elimination of some specific microorganisms. Functional pathways involved in fatty acid and sugar metabolism were affected by MF addition; meanwhile, the biosynthesis of SCFAs by gut microbiota was also enhanced. Second, we observed reduced liver fat accumulation in laying hens supplemented with MF, which could contribute to the changes in enzyme activities of lipid metabolism in the liver and variations in gut bacterial composition through the enterohepatic axis response. Moreover, dietary MF also showed the ability to improve the egg quality of laying hens, as indicated by an increase in PUFA content. This high egg quality was shaped by the active lipid metabolism in the liver and reduced gut microbial diversity of laying hens induced by dietary MF. The findings provide novel insights into the roles of the enterohepatic axis in the physiological state of laying hens and emphasize the potential application of MF as a feed additive in hen breeding.

### Supplementary Information

**Additional file 1: Table S1.** Composition and nutrient level in diet. **Table S2.** Fiber composition of Mulberry branch (GB5009.88–2014). **Figure S1.** Relative abundance of dominant bacterial phyla in the caecum digesta of laying hens with and without dietary MF. **Figure S2.** Relative abundance of dominant bacterial genera in the caecum digesta of laying hens with and without dietary MF. **Figure S3.** Variations in the relative abundance of dominant bacterial genera in the caecum digesta of laying hens with and without dietary MF. Different lowercases letters in each box of the same sub-figure represent significant differences among laying hens in different groups (Tukey's HSD test, *p* < 0.05). **Figure S4.** Relative abundance of dominant viral orders in the caecum digesta of laying hens with and without dietary MF. **Figure S5.** Relative abundance of dominant viral phyla in the caecum digesta of laying hens with and without dietary MF. **Figure S6.** Variations in the relative abundance of dominant viral genera in the caecum digesta of laying hens with and without dietary MF. Different lowercases letters in each box of the same sub-figure represent significant differences among laying hens in different groups (Tukey's HSD test, *p* < 0.05).

## Data Availability

The raw sequencing data have been deposited in the China National GeneBank Sequence Archive (CNSA) of the China National GeneBank DataBase (CNGBdb) with the accession number CNP0004855 (the review link: https://db.cngb.org/search/project/CNP0004855/).
