# Supplementary material for: Mulberry branch fiber improved lipid metabolism and egg yolk fatty acid composition of laying hens via the enterohepatic axis
Source: Microbiome. 2024 Apr 12;12:73. doi: 10.1186/s40168-024-01788-y (PMC11010431; doi:10.1186/s40168-024-01788-y)
Supplement: Supplementary file 2 — Additional file 1: Table S1. Composition and nutrient level in diet. Table S2. Fiber composition of Mulberry branch (GB5009.88–2014). Figure S1. Relative abundance of dominant bacterial phyla in the caecum digesta of laying hens with and without dietary MF. Figure S2. Relative abundance of dominant bacterial genera in the caecum digesta of laying hens with and without dietary MF. Figure S3. Variations in the relative abundance of dominant bacterial genera in the caecum digesta of laying hens with and without dietary MF. Different lowercases letters in each box of the same sub-figure represent significant differences among laying hens in different groups (Tukey's HSD test, p < 0.05). Figure S4. Relative abundance of dominant viral orders in the caecum digesta of laying hens with and without dietary MF. Figure S5. Relative abundance of dominant viral phyla in the caecum digesta of laying hens with and without dietary MF. Figure S6. Variations in the relative abundance of dominant viral genera in the caecum digesta of laying hens with and without dietary MF. Different lowercases letters in each box of the same sub-figure represent significant differences among laying hens in different groups (Tukey's HSD test, p < 0.05). [file 40168_2024_1788_MOESM1_ESM.docx]

**Supplementing information for**

#### **Mulberry branch fiber improved lipid metabolism and egg yolk fatty acid composition of laying hens via the enterohepatic axis**

Hong Hu ^1†^, Anjian Li ^1†^, Changyou Shi ^2†^, Liang Chen ^3^, Zelong Zhao ^4^, Xiaojian Yin ^1^, Qiang Zhang ^5^, Ying Huang ^1, *^, Hongbin Pan ^1, *^

^1^ Yunnan Provincial Key Laboratory of Animal Nutrition and Feed Science, Faculty of Animal Science and Technology, Yunnan Agricultural University, Kunming, 650201, China

^2^ University of Maryl and School of Medicine, Baltimore, MD 21228, USA

^3^ State Key Laboratory of Animal Nutrition, Institute of Animal Sciences, Chinese Academy of Agriculture Sciences, Beijing, 100193, China

^4^ Shanghai BIOZERON Biotechnology Co., Ltd., Shanghai, 201800, China

^5^ WOD Poultry Research Institute, Beijing, 100193, China

^†^ Hong Hu, Anjian Li, and Changyou Shi contributed equally to this work.

* Corresponding author: hying_5@163.com (Y. Huang); ynsdyz@163.com (H.B. Pan)

**Table S1** Composition and nutrient level in diet

| Ingredients (%) | CK | MF2 | MF3 | MF4 | MF5 |
| --- | --- | --- | --- | --- | --- |
| Corn | 61.60 | 60.10 | 58.20 | 56.22 | 54.28 |
| Soybean meal | 24.70 | 25.51 | 25.75 | 26.03 | 26.30 |
| Wheat bran | 2.00 | 0.00 | 0.00 | 0.00 | 0.00 |
| Mulberry branch powder | 0.00 | 2.00 | 3.00 | 4.00 | 5.00 |
| Soybean oil | 0.60 | 1.26 | 1.92 | 2.62 | 3.29 |
| CaHPO_4_·2H_2_O | 0.80 | 0.85 | 0.85 | 0.85 | 0.85 |
| Limestone | 9.30 | 9.28 | 9.28 | 9.28 | 9.28 |
| Premix^1^ | 1.00 | 1.00 | 1.00 | 1.00 | 1.00 |
| Total | 100.00 | 100.00 | 100.00 | 100.00 | 100.00 |
| Nutrition level (%) |  |  |  |  |  |
| Metabolic energy (MJ/kg) | 11.09 | 11.09 | 11.09 | 11.09 | 11.09 |
| Crude Protein | 15.86 | 15.86 | 15.86 | 15.86 | 15.86 |
| Ca | 3.67 | 3.67 | 3.67 | 3.67 | 3.68 |
| P | 0.52 | 0.51 | 0.51 | 0.51 | 0.50 |
| Lys | 0.80 | 0.81 | 0.81 | 0.81 | 0.81 |
| Met+Cys | 0.52 | 0.51 | 0.51 | 0.50 | 0.50 |

Note: ^1^ Premixes are available per kg of feeding: VA 8000-10000 IU, VD3 2200-5000 IU, VE 13 IU, VK3 1.4-4.8 mg, VB1 1.8 mg, VB2 3.0 mg, VB6 2.0 mg, VB12 0.01 mg, Niacinamide 20 mg, D-sodium pantothenate 10 mg, folic acid 0.55 mg, D-biotin 0.15 mg, choline 380 mg, Fe 60 mg, Cu 8 mg, Mn 60 mg, Zn 60 mg, I 0.35 mg, Se 0.12-0.48 mg. 2. nutrient level is calculated.

**Table S2.** Fiber composition of Mulberry branch (GB5009.88-2014).

| **Composition** | **Content** |
| --- | --- |
| Total dietary fiber (TDF) | 72.86% |
| Soluble dietary fiber (SDF) | 0.02% |
| Insoluble dietary fiber (IDF) | 72.87% |
| Protein | 4.43% |
| Ash | 11.18% |

**Figure S1.** Relative abundance of dominant bacterial phyla in the caecum digesta of laying hens with and without dietary MF.

**Figure S2.** Relative abundance of dominant bacterial genera in the caecum digesta of laying hens with and without dietary MF.

**Figure S3.** Variations in the relative abundance of dominant bacterial genera in the caecum digesta of laying hens with and without dietary MF. Different lowercases letters in each box of the same sub-figure represent significant differences among laying hens in different groups (Tukey's HSD test, p < 0.05).

**Figure S4.** Relative abundance of dominant viral orders in the caecum digesta of laying hens with and without dietary MF.

**Figure S5.** Relative abundance of dominant viral phyla in the caecum digesta of laying hens with and without dietary MF.

**Figure S6.** Variations in the relative abundance of dominant viral genera in the caecum digesta of laying hens with and without dietary MF. Different lowercases letters in each box of the same sub-figure represent significant differences among laying hens in different groups (Tukey's HSD test, p < 0.05).
